# Neutrophil extracellular DNA traps promote pancreatic cancer cells migration and invasion by activating EGFR/ERK pathway

**DOI:** 10.1111/jcmm.16555

**Published:** 2021-05-06

**Authors:** Wei Jin, Huijing Yin, Hao Li, Xian‐Jun Yu, Hua‐Xiang Xu, Liang Liu

**Affiliations:** ^1^ Shanghai Institute of Immunology Department of Immunology and Microbiology Key Laboratory of Cell Differentiation and Apoptosis of Chinese Ministry of Education Shanghai Jiao Tong University School of Medicine Shanghai China; ^2^ Department of Pancreatic Surgery Pancreatic Cancer Institute Fudan University Shanghai Cancer Center Department of Oncology Shanghai Medical College Shanghai China; ^3^ Translational Medicine Center Shanghai General Hospital Shanghai Jiao Tong University School of Medicine Shanghai China

**Keywords:** EMT, IL‐1β, NETs, Pancreatic Cancer

## Abstract

Neutrophil extracellular DNA traps (NETs) are newly discovered forms of activated neutrophils. Increasing researches have shown that NETs play important roles in cancer progression. Our previous study has proved that tumour‐infiltrating NETs could predict postsurgical survival in patients with pancreatic ductal adenocarcinoma (PDAC). However, the roles of NETs on the progression of pancreatic cancer are unknown. Here, we investigated the effects of NETs on pancreatic cancer cells. Results showed that both PDAC patients’ and normal individuals’ neutrophils‐derived NETs could promote migration and invasion of pancreatic cancer cells with epithelial‐mesenchymal transition. Further, study confirmed that EGFR/ERK pathway played an important role in this progression. The addition of neutralizing antibodies for IL‐1β could effectively block the activation of EGFR/ERK companied with reduction of EMT, migration and invasion. Taken together, NETs facilitated EMT, migration and invasion via IL‐1β/EGFR/ERK pathway in pancreatic cancer cells. Our study suggests that NETs may provide promising therapeutic targets for pancreatic cancer.

**Highlights**

- NETs could promote migration and invasion of pancreatic cancer cells.
- Both NETs derived from neutrophils of PDAC patients and normal individuals could promote the migration and invasion of pancreatic cancer cell.
- NETs promote PDAC cells migration, invasion and EMT which is dependent on IL‐1β/EGFR/ERK pathway.
- NETs correlate with EMT markers in PDAC patient's tissue.

## 1 INTRODUCTION

Patients with pancreatic ductal adenocarcinoma (PDAC) have an extremely poor prognosis, with a 5‐year overall survival rate of less than 5.0%.^1^ Pancreatic cancer has a higher mortality rate due the high incidence of recurrence and metastases dissemination.^2^ Thus, figuring out the mechanisms regulating pancreatic metastasis is essential to treat high‐risk patients.

Carcinogenesis is closely linked to a dysfunctional immune system.^3^ The interplay between cancer and the immune system may influence many aspects of cancer progression.^4^ Neutrophils are generally the most abundant immune cell population and involved in the development of cancer.^5^ Recent studies suggest that high peripheral blood neutrophil‐to‐lymphocyte ratio is an indicator of poor survival in patients with pancreatic cancer.^6^ Moreover, tumour‐infiltrating neutrophils are frequently observed in patients with pancreatic cancer and are associated with reduced survival.^7^

Under specific stimulatory conditions such as infection and reactive oxygen species, neutrophils release chromatin structures which are decorated with specific cytoplasmic and granular proteins. These structures then form DNA meshes; special forms of neutrophils called neutrophil extracellular DNA traps (NETs). As a part of innate immunity in humans, neutrophils release decondensed chromatin networks to capture and disarm bacteria and fungi via NETosis. Citrullination of histone H3 (Cit‐H3) by peptidylarginine deiminase 4 is an important step in NETosis.^8^ In addition, the activity of myeloperoxidase (MPO) is critical in the process of DNA extrusion. Both Cit‐H3 and MPO serve as markers for formation of NETs.^9^ NETs play important roles in the innate immune response, pathologic alteration and development of cancer progression.^10^ It has been reported that breast cancer cells can induce metastasis‐supportive NETs.^11^ NETs could promote cancer metastasis by sequestering and entrapping circulating cancer cells through interactions induced by b1‐integrin.^12^ NETs formation is conducive for implantation of ovarian cancer cells, and blockade of NETs formation could prevent omental metastasis.^13^ NETs could act as an adhesion substrate for different tumour cells.^14^ NETs also act as a drug target to counteract chronic and acute inflammation.^15^ It has also been reported that NETs could promote EMT^16^ and enhance metastasis through CCDC25^17^ in breast cancer.

The high mortality of PDAC is attributed to its aggressive biological phenotype, which is characterized by frequent invasion and metastasis.^18^ 80% of pancreatic cancer patients are diagnosed with metastasis, and about 50% of patients suffer liver metastasis within 2 years after receiving surgery and adjuvant therapy. Evolutionary biological process called epithelial‐to‐mesenchymal transition (EMT) contributes to pancreatic metastatic dissemination.^19^ During the process of pancreatic cancer EMT, epithelial marker proteins, such as E‐cadherin^20^ and ZO‐1,^21^ decline. And the expression levels of mesenchymal markers N‐cadherin^22^ and vimentin^23^ increase. EMT is regulated by complex regulatory networks in pancreatic cancer by EMT‐related transcription factors, especially SNAIL (zinc finger protein SNAIL),^20^ ZEB (Zinc finger E‐box binding homeobox 1)^24^ and Twist (twist basic helix‐loop‐helix transcription factor 1).^25^ Matrix metalloprotein such as MMP2 and MMP16 also plays important roles in pancreatic EMT.^26^

Our previous studies have shown that NETs are associated with poor prognosis and high frequency of metastasis in patients with PDAC.^27^, ^28^ The specific mechanism of NETs promoting the invasion and metastasis of pancreatic cancer has not been clearly studied. Our study shows that NETs promote PDAC migration, invasion and EMT through IL‐1β/EGFR/ERK pathway. And inhibition of IL‐1β/EGFR/ERK pathway could effectively inhibit NETs‐induced migration, invasion and EMT of pancreatic cancer.

## 2 MATERIALS AND METHODS

### 2.1 Cell culture

BxPC3, MIA PaCa2 and PANC1 cell lines were provided by the Cell Bank, Type Culture Collection, Chinese Academy of Sciences (Shanghai, China). BxPC3 was maintained in RPMI 1640 media (Corning, NY, USA) with 10% foetal bovine serum (Gibco, Grand Island, NY, USA). And, MIA PaCa2 was cultured in DMEM containing 10% FBS (Gibco, Grand Island, NY, USA). All medium were supplemented with 100 μg/mL streptomycin and 100 U/mL penicillin. All pancreatic cancer cell lines were cultured in a humidified atmosphere containing 5% CO_2_ at the temperature of 37°C.

### 2.2 Patients and specimens

Patients with PDAC were subjected to the following inclusion conditions: (a) complete follow‐up data, (b) had undergone R0 resection for PDAC that was identified by two experienced pathologists, (c) no history of another malignant tumour, (d) no history of any antitumour treatment and (e) no death within 30 days of surgery caused by postoperative complications. We totally involved 150 patients in our experiments.

The Research Ethics Committees of Fudan University and the Pancreatic Center approved the use of human tissues. Informed consents were obtained from all patients according to the regulations. The associated permit number is 050432‐4‐1212.

Immunohistochemical staining was evaluated by two independent pathologists who were blinded to the clinical data. The intensity of the staining was graded as 0, 1 or 2. The percentage of positive cells was graded as 0 (0%), 1 (≤1%), 2 (≤5%), 3 (≤10%), 4 (≤20%) or 5 (>20%). The two grades were added together. A total score of 0 to 1 was considered as low expression. A total score of 2‐5 was considered as a median expression. A total score of 6 to 7 was considered as high expression.

### 2.3 Conditioned medium (CM)

We extracted neutrophils from the blood, inoculated them in 5% RPMI medium which was made by supplementing RPMI media with 5% foetal bovine serum (FBS) at the concentration of 5 × 10^6^ neutrophils/ml. Stimulate neutrophils with 5 nM of PMA for 8hr at 37°C 5% CO2. This will allow NETosis. Collected conditioned medium (CM) was centrifuged at 2000rp/min and filtered through 0.2um filter to remove the DNA meshes of NETs. Collected conditioned medium (CM) was concentrated using an Amicon Ultra Centrifugal Filter device (Merck Millipore) with a molecular weight cut‐off of 10 kDa. 10 ml conditioned media were concentrated to 0.5 ml. We stored the CM at –80°C until use. The CM was added into the culture medium at a 1:10 ratio.

### 2.4 Drugs and antibodies

The antibodies used were anti‐phospho‐EGFR1068 (Cell Signaling Technology, #3777), anti‐EGFR (Cell Signaling Technology, #4267), anti‐ERK (Cell Signaling Technology, #4695), anti‐phospho‐ERK (Cell Signaling Technology, # 4370), anti‐Snail (Cell Signaling Technology, #3879), anti‐E‐cadherin (Cell Signaling Technology, #14472), anti‐vimentin (Cell Signaling Technology, # 5741), anti‐N‐cadherin (Cell Signaling Technology, #13116), anti‐IL‐1β (Cell Signaling Technology, #12703), anti‐Cit‐H3 (Abcam, ab5103), anti‐H3 antibodies (Abcam, ab1791) and anti‐β‐actin antibody (Abcam, ab8226). The inhibitors erlotinib, SHC772984 and Sivelestat (ONO‐5046) were purchased from Selleck Chemicals (Selleck Chemicals, Houston, TX, USA).

### 2.5 Real‐time PCR

Total RNA was extracted using the TriPure Isolation reagent (Roche). Complementary DNA was obtained using the M‐MLV Reverse Transcriptase Synthesis kit (Promega). qPCR was subsequently carried out using the Power SYBR Green PCR Master Mix kit (Applied Biosystems). Relative quantities were calculated using the ΔΔCt method and the endogenous reference β‐actin. Each experiment was performed in triplicate.

Primer sequences for qPCR.

**Table:** 

Gene	Sequence
Snail Up	5'‐ACTGCGACAAGGAGTACACC‐3'
Snail Down	5'‐GAGTGCGTTTGCAGATGGG‐3'
Slug Up	5'‐CGAACTGGACACACATACAGTG‐3'
Slug Down	5'‐CTGAGGATCTCTGGTTGTGGT‐3'
Twist Up	5'‐GCCTAGAGTTGCCGACTTATG‐3'
Twist Down	5'‐TGCGTTTCCTGTTAAGGTAGC‐3'
Zeb1 Up	5'‐GATGATGAATGCGAGTCAGATGC‐3'
Zeb1 Down	5'‐ACAGCAGTGTCTTGTTGTTGT‐3'
Zeb2 Up	5'‐CAAGAGGCGCAAACAAGCC‐3'
Zeb2 Down	5'‐GGTTGGCAATACCGTCATCC‐3'
E‐Cadherin Up	5'‐CGAGAGCTACACGTTCACGG‐3'
E‐Cadherin Down	5'‐GGGTGTCGAGGGAAAAATAGG‐3'
N‐Cadherin Up	5'‐TCAGGCGTCTGTAGAGGCTT‐3'
N‐Cadherin Down	5'‐ATGCACATCCTTCGATAAGACTG‐3'
Vimentin Up	5'‐GACGCCATCAACACCGAGTT‐3'
Vimentin Down	5'‐CTTTGTCGTTGGTTAGCTGGT‐3'
IL‐6 Up	5'‐GAAGAGCGCCGCTGAGAAT‐3'
IL‐6 Down	5'‐GTGCAGAGGGTTTAATGTCAACT‐3'
IL‐1β Up	5'‐ATGATGGCTTATTACAGTGGCAA‐3'
IL‐1β Down	5'‐GTCGGAGATTCGTAGCTGGA‐3'
IL‐1A Up	5'‐TGGTAGTAGCAACCAACGGGA‐3'
IL‐1A Down	5'‐ACTTTGATTGAGGGCGTCATTC‐3'
IL‐7A Up	5'‐TTGGACTTCCTCCCCTGATCC‐3'
IL‐7A Down	5'‐TCGATGCTGACCATTAGAACAC‐3'
IL‐17A Up	5'‐TCCCACGAAATCCAGGATGC‐3'
IL‐17A Down	5'‐GGATGTTCAGGTTGACCATCAC‐3'
IL‐4 Up	5'‐CCAACTGCTTCCCCCTCTG‐3'
IL‐4 Down	5'‐TCTGTTACGGTCAACTCGGTG‐3'
IL‐11 Up	5'‐CGAGCGGACCTACTGTCCTA‐3'
IL‐11 Down	5'‐GCCCAGTCAAGTGTCAGGTG‐3'
IL‐33 Up	5'‐GTGACGGTGTTGATGGTAAGAT‐3'
IL‐33 Down	5'‐AGCTCCACAGAGTGTTCCTTG‐3'
IL‐17F Up	5'‐GCTGTCGATATTGGGGCTTG‐3'
IL‐17F Down	5'‐GGAAACGCGCTGGTTTTCAT‐3'
IL‐37 Up	5'‐TTCTTTGCATTAGCCTCATCCTT‐3'
IL‐37 Down	5'‐CGTGCTGATTCCTTTTGGGC‐3'
TNF‐α Up	5'‐CCTCTCTCTAATCAGCCCTCTG‐3'
TNF‐α Down	5'‐GAGGACCTGGGAGTAGATGAG‐3'
IL‐10 Up	5'‐GACTTTAAGGGTTACCTGGGTTG‐3'
IL‐10 Down	5'‐TCACATGCGCCTTGATGTCTG‐3'
IL‐36 Up	5'‐TCCCGAAGAGTTGTGTTTTGG‐3'
IL‐36 Down	5'‐TGAGTGTGTCAGTATGGCTTGA‐3'
IL‐29 Up	5'‐CACATTGGCAGGTTCAAATCTCT‐3'
IL‐29 Down	5'‐CCAGCGGACTCCTTTTTGG‐3'
IL‐14 Up	5'‐CTCAAGCCAGAACGGCTCAG‐3'
IL‐14 Down	5'‐TCCATTGACTACTGGAGTTGGTT‐3'
β‐actin Up	5'‐CATGTACGTTGCTATCCAGGC‐3'
β‐actin Down	5'‐CTCCTTAATGTCACGCACGAT‐3'

### 2.6 Western blot

Cells were collected and lysed in NETN 150 lysis buffer. The quantities of proteins were identified using the Bradford assay. 20 μg protein was then separated by SDS‐PAGE and transferred to a nitrocellulose membrane (Axygen, Tewksbury, MA, USA). The membrane was incubated with 5% non‐fat milk for 1 h at room temperature and then with the indicated primary antibodies (1:1000) at 4°C for 12 h. The membrane was then incubated with HRP‐conjugated secondary antibodies (1:1000). We used the Millipore Immobilon Western Chemiluminescent HRP Substrate kit to detect the protein expression.

### 2.7 Cell proliferation assay

Cell proliferation was determined using the cell counting kit 8 (Dojindo Molecular Technologies, Kumamoto, Japan).^29^ Each experiment was repeated three times with triplicate samples.

### 2.8 Immunofluorescence staining

4% cold formaldehyde was used for the fixation of NETs, and then NETs were washed twice with PBS. NETs were incubated with Cit‐H3 antibody (diluted 1:100, anti‐histone H3, ab5103) at 4°C for 12 h and then blocked with 5% BSA. The slices were incubated with fluorescent secondary antibodies (Jackson ImmunoResearch, West Grove, PA, USA) diluted in 5% BSA (Jackson ImmunoResearch) for 60 min at 37°C. Finally, DAPI (Vector Laboratories, Burlingame, CA, USA) was used to stain nuclei. The slices were captured by confocal microscopy (Nikon, Tokyo, Japan).

### 2.9 Migration and invasion assays

BxPC3 and MIA PaCa2 cells were seeded into 6‐well plates and cultured for 12 h to achieve 100% confluence. A scratch was made in each well by a needle followed by two washes with PBS. Then, the cells were incubated in serum‐free media for 48 h. Photographs were taken by light microscopy immediately after the scratch (0 h) and 48 h after the scratch (48 h). For the wound healing assays, BxPC3 and MIA PaCa2 were treated with 10 μg/mL mitomycin C for 4 h to inhibit proliferation prior to the experiments.

Pancreatic cancer cells (1 × 10^5^) were added to the upper inserts of the Transwell plate (Corning, 8 μm) coated with Matrigel (BD Biosciences, San Jose, CA, USA) in serum‐free media, and CM was added to the lower chamber. The CM‐neutrophils or CM‐NETs were derived from neutrophil cultures that were treated with or without 5 nM phorbol 12‐myristate 13‐acetate (PMA; Selleck). Sivelestat was then added to block the formation of NETs. After incubation at 37°C for 48 h, non‐invasive cancer cells were removed from the upper surface of the membrane, and the bottom side of the membrane was fixed with 4% formaldehyde. The membrane was then stained with crystal violet. The invading cells were counted, and the number of cells averaged over six fields of view. For the cell migration and invasion assays, BxPC3 and MIA PaCa2 were treated with 10 μg/mL mitomycin C for 4 h to inhibit proliferation prior to the experiments.

### 2.10 Animal experiments

All animals were treated under the Guide for the Care and Use of Laboratory Animals and the Principles for the Utilization and Care of Vertebrate Animals. The group allocation was blinded to the investigators. Ventral flanks of female BALB/c nude mice were injected with 2.5 × 10^6^ MIA PaCa2 cells subcutaneously. When the tumours reached approximately 4 mm ×4 mm in size, tumour‐bearing mice were randomly assigned to different experimental groups (n = 4 per group) and treated with different types of conditioned medium. We seeded neutrophils at a density of 2X10^6^/ml. The conditioned medium was collected 8 hours later with or without PMA treatment. Intratumoural injection of 500 µL CM was performed every day. The volume was calculated according to the formula: V = ½ × length × width^2^. All animal studies were approved by the Committee on the Use of Live Animals in Teaching and Research in Fudan University, and the associated permit number is JS‐060.

### 2.11 Statistical analysis

All statistical analyses were carried out using GraphPad Prism 6 software. Each result is shown as the mean ±SD of three independent experiments (n = 3). The statistical significance of comparisons among multiple groups was assessed by one‐way analysis of variance, followed by Bonferroni analysis, with *P* <.05 considered statistically significant. Correlation between two groups was assessed by Pearson's correlation analysis. *P* value less than 0.05 was considered statistically significant (*: 0.05, **: < 0.01 and ***: < 0.001).

## 3 RESULTS

### 3.1 NETs promote migration and invasion of pancreatic cancer cells

**FIGURE 1:**
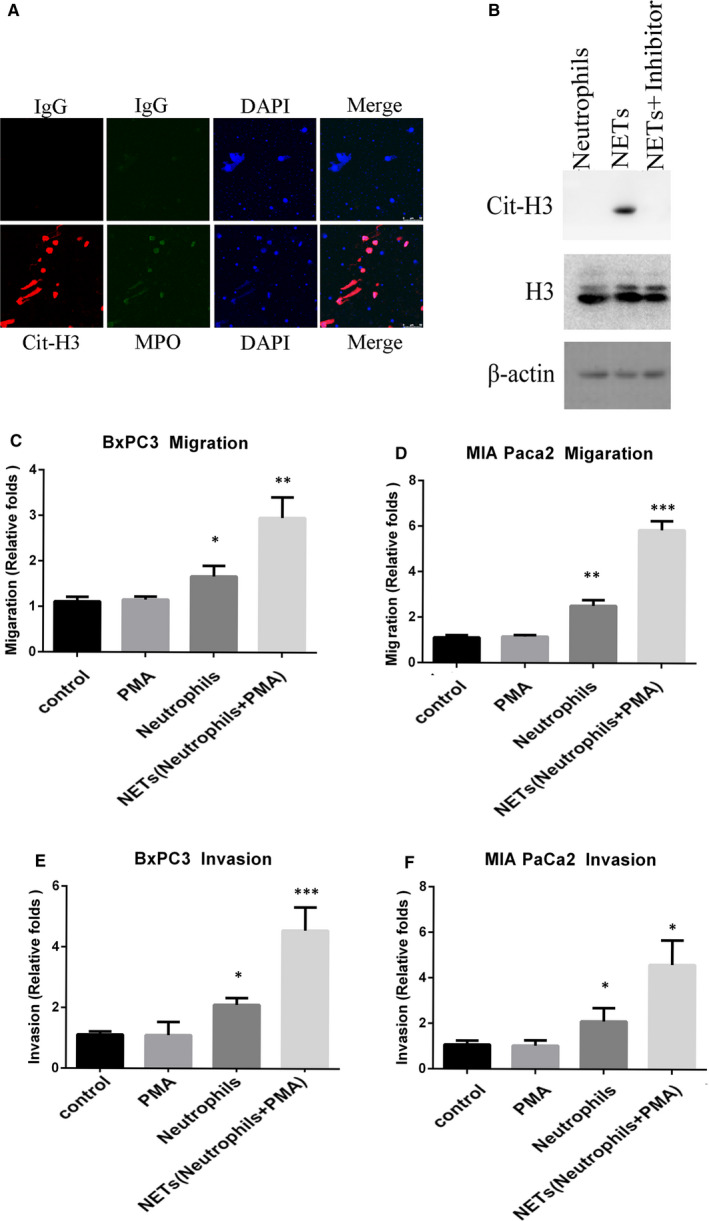
NETs enhance migration and invasion of pancreatic cancer cells. A, Neutrophils were extracted from patients’ blood and treated with 5 nM PMA for 8 h. Identification of NETs was carried out by immunofluorescence staining for MPO and Cit‐H3. B, Western blot analysis showed Cit‐H3 and H3 expression of neutrophils and NETs from PDAC patients. C and D, The migration of BxPC3 (C) and MIA PaCa2 (D) were determined using the wound healing assay. Cancer cells were cultured with neutrophils and CM‐NETs separately for 48 h (magnification, ×20). E and F, Transwell assays of BxPC3 (E) and MIA PaCa2 (F) cells. The CM‐neutrophils and CM‐NETs were added to the lower chamber. The number of cells passing through the upper chamber was counted in three fields after 48 h (magnification, ×20). Results are presented as the mean ±SD

To investigate the influence of NETs on pancreatic cancer cells, we isolated neutrophils from patient blood and induced the formation of NETs in vitro by PMA treatment. We examined MPO and Cit‐H3 levels by immunofluorescence to verify the formation of NETs (Figure 1A). To confirm the specificity of NETs, we added NETs inhibitors sivelestat to inhibit NETs formation. The results show that NETs inhibitor can effectively inhibit the formation of Cit‐H3, indicating the specificity of NETs formation (Figure 1B).

One of the most significant features of PDAC is the abundant stroma.^27^ Our previous study has also showed that NETs were mainly detected in the intratumoural stroma rather than tumour nests in a diffused manner in pancreatic cancer tissue. Due to the abundant stroma, NETs were in a distance to the pancreatic ductal cancer cells. The related findings have been published in Annals of Surgical Oncology.^27^, ^28^ In consideration of that there were no direct interactions and co‐location in PDAC cancer tissue, we think NETs could perform important functions without direct contact with tumour cells. Thus, we treated BxPC3, MIA PaCa2 and PANC1 cells with conditioned medium of NETs (CM‐NETs) and performed experiments to identify the influence on proliferation, migration and invasion. Wound healing assay and transwell assay showed that NETs conditioned medium increased both the migratory and invasive abilities of BxPC3 and MIA PaCa2 cells without influencing proliferation (Figure 1C–F, Figure S1A, B).

**FIGURE 2:**
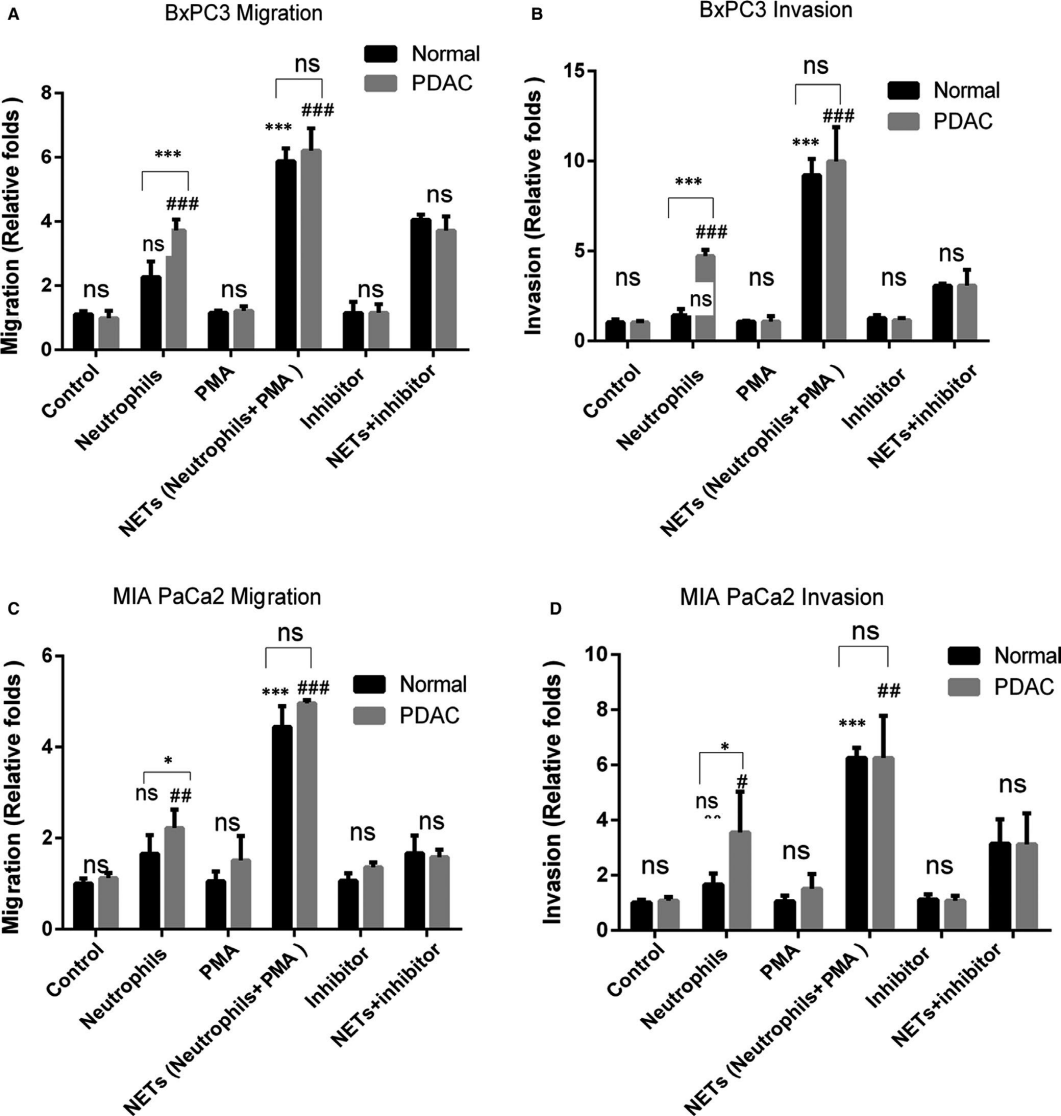
Both NETs from patients and normal individuals could promote the migration and invasion of pancreatic cancer. Neutrophils were extracted from the blood of patients and normal individuals to induce the formation of NETs. PMA was used to induce NETs formation. Sivelestat (40 nM) was used to inhibit the formation of NETs. The wound healing assay and invasion assays were carried out with the indicated CM. The CM‐neutrophils and CM‐NETs were added to the lower chamber. BxPC3 or MIA PaCa2 cells were added to the upper chamber in absence of serum. The number of cells passing through the upper chamber was counted in three fields after 48 h (magnification, ×20). A‐B;Results of migration and invasion of BxPC3 were shown. C‐D;Results of migration and invasion of MIA PaCa2 were shown. Results are presented as the mean ±SD

As previous studies reported that neutrophils could be divided into anti‐tumourigenic N1 phenotype and pro‐tumourigenic N2 phenotype basing on their function. Until now, no specific marker has been identified to distinguish the N1/N2 subgroups in clinical research and diagnosis.^27^ We wanted to investigate whether there was any difference in the function of neutrophils and NETs derived from PDAC patients and normal donors. Furthermore, our study found that patient‐derived neutrophils promoted the migration and invasion of pancreatic cancer cells significantly, whereas neutrophils derived from normal individuals could not (Figure 2A‐D). It suggested that neutrophils from PDAC tended to exhibit pro‐tumourigenic phenotype while neutrophils from normal did not. The function of neutrophils dependent on its origin. Interestingly, both NETs induced from neutrophils of normal donors and pancreatic cancer patients could promote the migration and invasion of pancreatic cancer cells without difference (Figure 2A‐D). The same findings were also verified in PANC1 cells (Figure S2A and B). There was no difference in the function of NETs derived from PDAC patients or normal donors. The specific mechanism is important for pancreatic cancer research and needs further study.

### 3.2 NETs induce EMT in pancreatic cancer cells

One of important process in cancer metastasis is EMT, by which cancer cells lose their epithelial characteristics and exhibit mesenchymal‐like features with high migratory and invasive capacities.^30^

**FIGURE 3:**
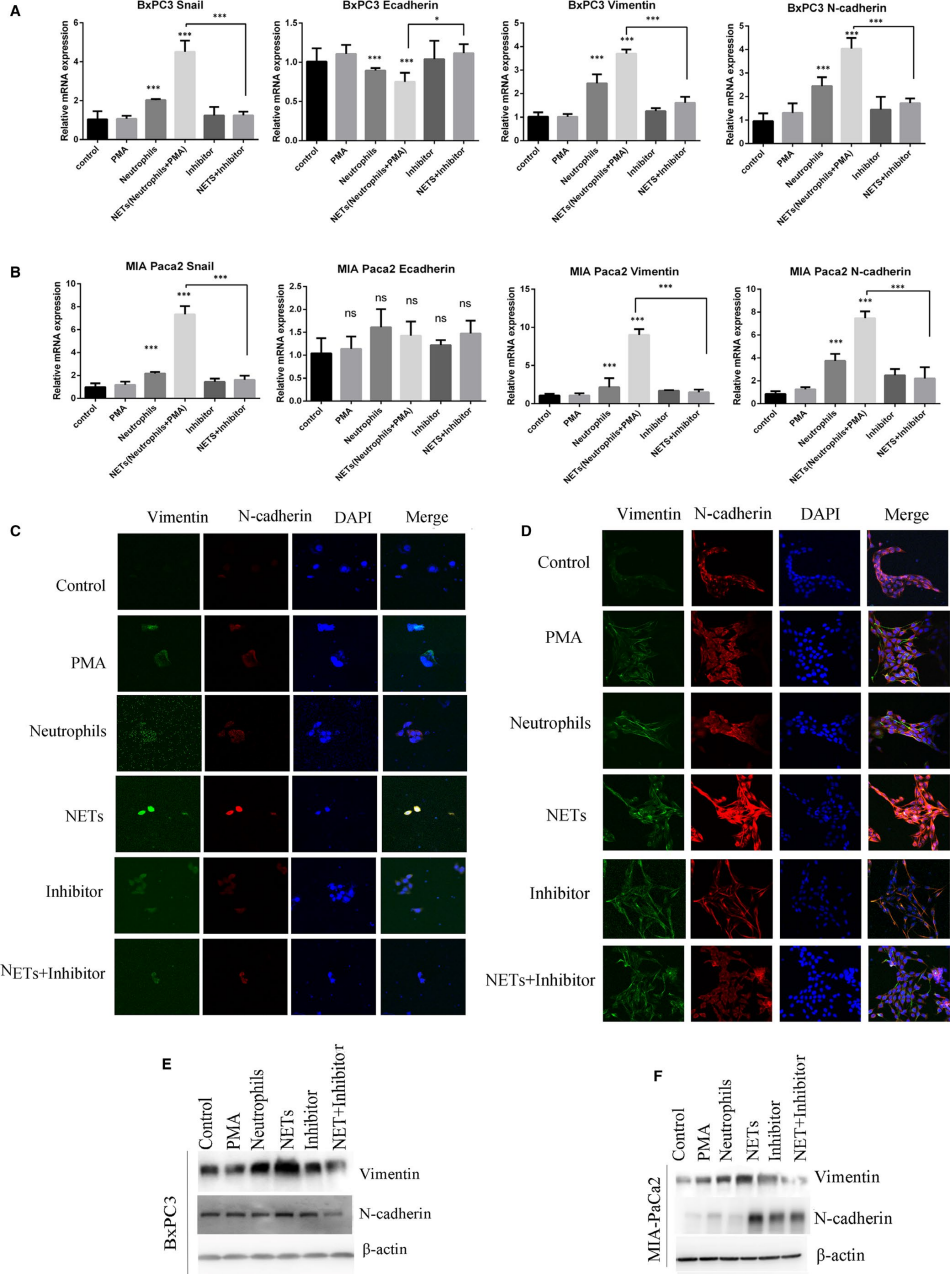
NETs promote EMT in pancreatic cancer. A and B. Pancreatic cancer cells were treated with conditioned medium for 24 h. Real‐time PCR was used to assess the expression of the EMT‐related genes in BxPC3 (A) and MIA PaCa2 (B) cells after the specified treatment. C and D, Vimentin (green) and N‐cadherin (red) were detected by immunofluorescence in BxPC3(C) and MIA PaCa2 (D) after the specified treatment. Nuclei were stained with DAPI (blue). E and F, Western blot analysis of the EMT‐related proteins in BxPC3 (E) and MIA PaCa2 (F) after the specified treatment. PMA was used to induce NETs formation. Sivelestat (40 nM) was used to inhibit the formation of NETs

To determine whether NETs could influence EMT, we treated cancer cells with NETs and measured the levels of various epithelial and mesenchymal markers. The Real‐time PCR results showed that treatment with CM‐NETs significantly increased the mRNA levels of Snail, N‐cadherin and vimentin, which are markers of mesenchymal cells, and reduced the mRNA levels of E‐cadherin, which is an epithelial cell marker (Figure 3A‐B, Figure S1C‐K, Figure S2C–F). Immunofluorescence and Western blot analysis results showed upregulated protein levels of vimentin and N‐cadherin in BxPC3 and MIA PaCa2 cells after treatment with CM‐NETs (Figure 3C‐F). These results collectively confirmed that NETs could promote EMT in pancreatic cancer cells.

### 3.3 Tumour migration, invasion and EMT induced by NETs are dependent on EGFR/ERK signalling

To determine the mechanical link between NETs and EMT in pancreatic cancer cells, we investigated several important EMT relative pathways such as EGFR,^31^ WNT,^32^ TGF‐β,^33^ AKT^34^ and mTOR^35^ pathway.

**FIGURE 4:**
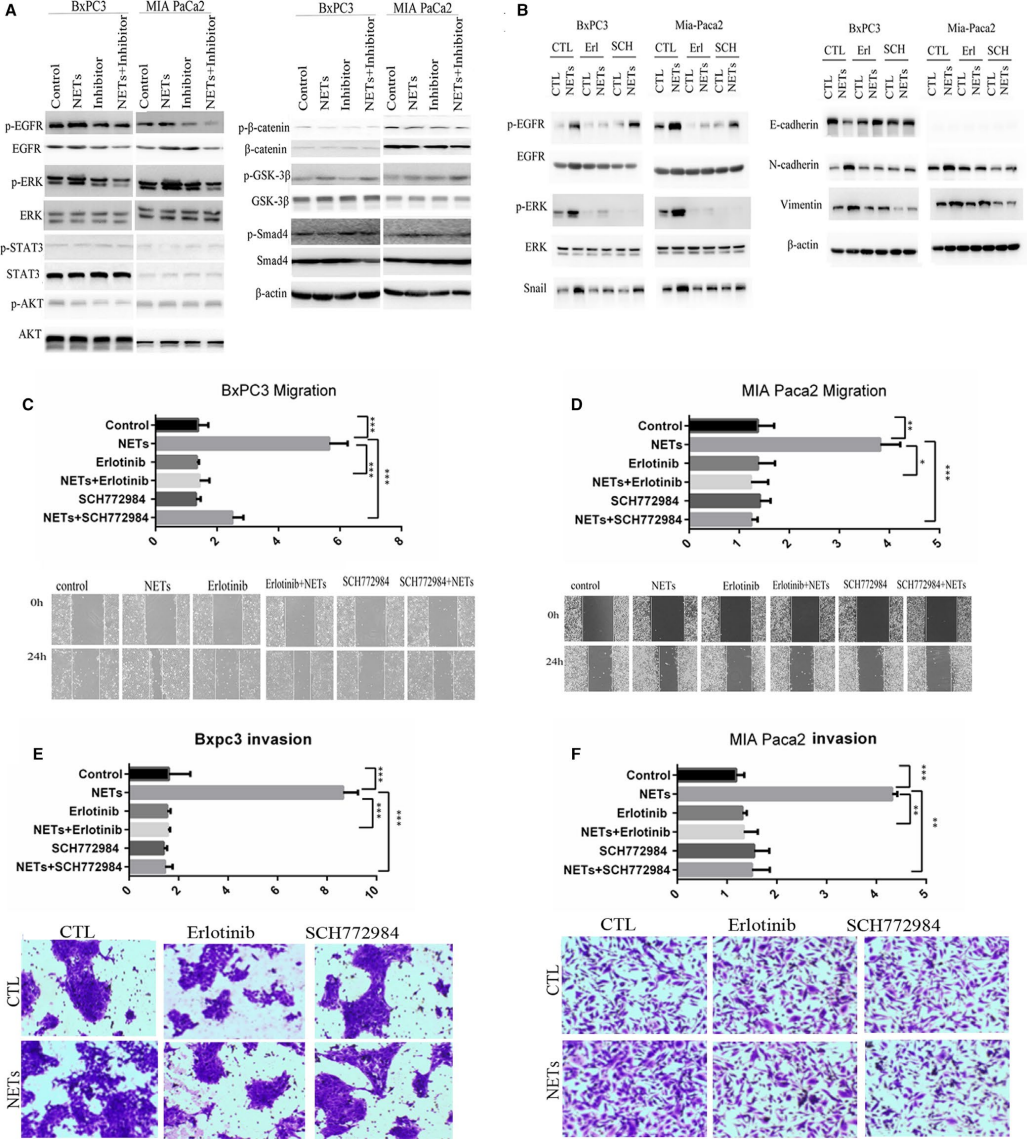
NETs promote pancreatic cancer EMT via EGFR/ERK‐dependent pathway. A, BxPC3 and MIA PaCa2 were cultured in indicated conditioned medium. Western blot analysis of the indicated proteins was performed in BxPC3 and MIA PaCa2. β‐actin was used as the loading control. B, BxPC3 and MIA PaCa2 were cultured in CM‐NETs and then treated with erlotinib (20 μM) or SCH772984 (10 nM) for 24 h. The expression levels of indicated proteins were analysed by Western blot analysis. C and D, The migratory abilities of BxPC3 and MIA PaCa2 were assessed using the wound healing assay. BxPC3 and MIA PaCa2 cells were cultured with CM‐neutrophils or CM‐NETs in the presence or absence of erlotinib (20 μM) or SCH772984 (10 nM) for 48 h. E and F, The invasive abilities of BxPC3 and MIA PaCa2 were determined using the Matrigel invasion assay. BxPC3 and MIA PaCa2 were cultured with CM‐neutrophils or CM‐NETs (added to the lower chamber) in the presence or absence of erlotinib (20 μM) or SCH772984 (10 nM) for 48 h

The results showed that NETs activated EGFR/ERK in both BxPC3 and MIA PaCa2, whereas the inhibition of NETs blocked the activation (Figure 4A). While other EMT‐related pathways such as WNT, PI3K and TGFβ were not affected by NETs in pancreatic cancer cells. Western blot results showed that NETs upregulated EGFR/ERK pathway specifically. Importantly, the inhibition of both EGFR (by the EGFR inhibitor erlotinib) and ERK (by the ERK inhibitor SCH772984) blocked the induction of EMT‐related genes by NETs (Figure 4B). All the results collectively suggested that EGFR/ERK pathway was important in NETs‐induced EMT.

To determine whether NETs mediated migration and invasion of pancreatic cancer cells are EGFR/ERK dependent, we examined the effects of EGFR and ERK inhibitors on pancreatic cancer cells after treatment with CM‐NETs. The results of wound healing assay and transwell assay showed that EGFR/ERK inhibition significantly reduced NETs‐induced pancreatic cancer cell migration and invasion without influencing proliferation (Figure 4C‐F, Figure S3A and B). Taken together, these results showed that NETs promoted EMT, migration and invasion via the EGFR/ERK pathway.

### 3.4 IL‐1β is the mediator of NETs‐induced migration, invasion and EMT

**FIGURE 5:**
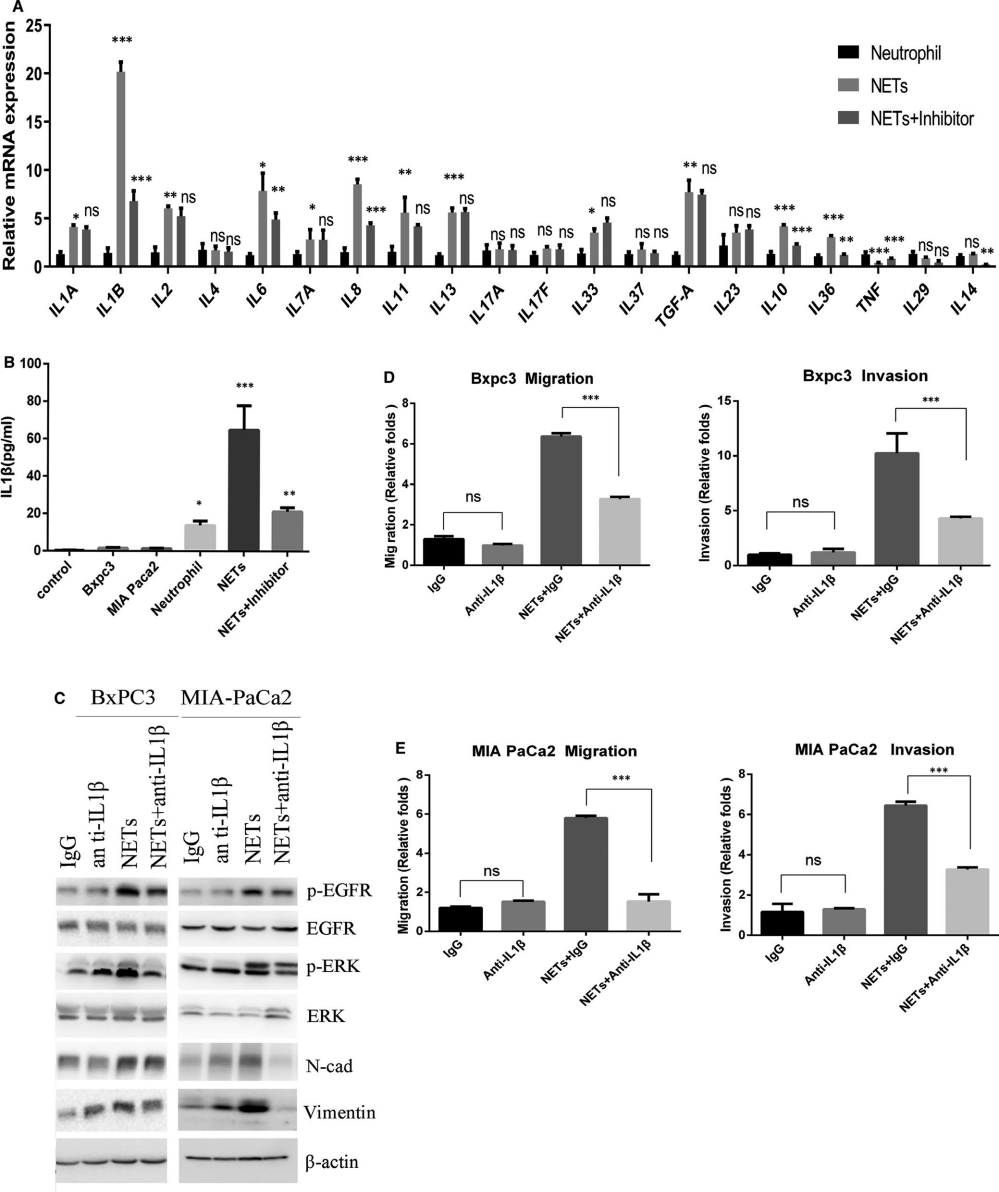
NETs promote pancreatic cancer migration, invasion and EMT via IL‐1β. A, Real‐time PCR analysis of the mRNA levels of indicated inflammatory cytokines. B. ELISA assay was used to measure IL‐1β levels secreted by different cells. C, Indicated proteins were analysed by Western blot analysis. D, Migratory abilities of BxPC3 and MIA PaCa2 were assessed using the wound healing assay. E, Matrigel invasion assay was carried out to identify the invasive abilities of BxPC3 and MIA PaCa2

Next, we examined the mechanism by which NETs could activate the EGFR pathway. It has been shown that various kinds of cytokines are secreted in the formation of NETs which maybe induce the invasive and migratory abilities of pancreatic cancer cells, such as IL‐6, IL‐1β, IL‐1A, IL‐7A, IL‐17A, IL‐4, IL‐11, IL‐33, IL‐17F, IL‐37, TGF‐α TNF, IL‐23, IL‐10, IL‐36, IL‐29 and IL‐14.^16^, ^36^, ^37^, ^38^, ^39^, ^40^ Real‐time PCR results showed that IL‐1β, IL‐6, IL‐8, IL‐10 and IL‐36 mRNA levels were significantly upregulated in NETs and downregulated when the formation of NETs was inhibited (Figure 5A). The data showed that NETs released more than 10 times of IL‐1β than neutrophils from PDAC patients. ELISA results also confirmed that almost all IL‐1β were from the secretion of NETs (Figure 5B). It has been reported that IL‐1β plays important roles in EGFR activation, such as IL‐1β transactivates EGFR via the CXCL1‐CXCR2 axis.^41^ It has also been shown that IL‐1β plays important roles in EMT^42^ and pancreatic cancer progression.^43^ While the influence of IL‐1β on EGFR has not been reported in PDAC, we performed more experiments to figure out the role of IL‐1β in NETs‐induced PDAC migration and invasion. To determine whether IL‐1β is the main factor in NETs‐induced EMT, we examined the effects of IL‐1β inhibition after treatment with anti‐IL‐1β antibodies. Western blot results showed that NETs‐induced activation of EGFR/ERK and the EMT biomarkers upregulation were significantly blocked by treatment with anti‐IL‐1β antibodies (Figure 5C, Figure S2I and S3C and D).

Moreover, inhibition of IL‐1β attenuated the migration and invasion of BxPC3, MIA PaCa2 and PANC1 mediated by NETs induced from PDAC patients’ neutrophils (Figure 5D, E, Figure S2G and H). To confirm the function and mechanism of NETs, we performed the following experiments by using NETs induced by neutrophils from normal. The results showed that neutrophils from normal did not influence the migration and invasion, while NETs could promote the migration and invasion of pancreatic cancer cells (Figure S4A–D). And inhibition of IL‐1β effectively blocked the migration and invasion induced by NETs derived from normal individual's neutrophils. The results confirmed that NETs could independently affect the migration and invasion of pancreatic cancer.

Taken together, these data suggest that IL‐1β is responsible for NETs‐induced pancreatic cancer cell mobility and EMT.

### 3.5 NETs correlate with EGFR activation and EMT in vivo

**FIGURE 6:**
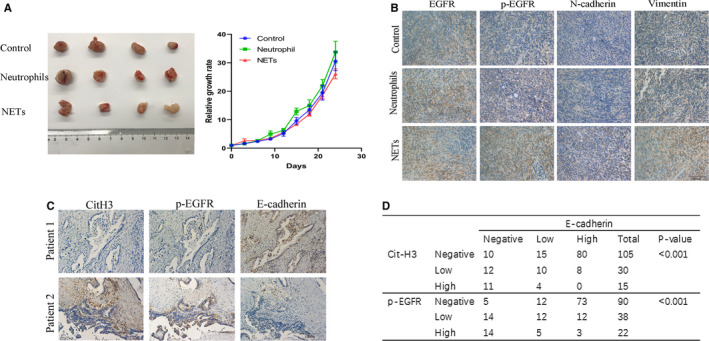
NETs promote EGFR‐dependent EMT in vivo. A. Ventral flanks of female BALB/c nude mice were injected with 2.5 × 10^6^ MIA PaCa2 cells subcutaneously. When the tumours reached approximately 4 × 4 mm in size, tumour‐bearing mice were randomly assigned to different experimental groups (n = 4 per group) and treated with different types of conditioned medium. We seeded neutrophils at a density of 5 × 10^6^/ml with or without PMA and collected the conditioned medium 8 h later. Collected conditioned medium (CM) was concentrated using an Amicon Ultra Centrifugal Filter device (Merck Millipore) with a molecular weight cut‐off of 10 kDa. 10 ml conditioned media were concentrated to 0.5 mL. Intratumoural injection of 500 µL concentrated CM was performed every day. The volume was calculated according to the formula: V = ½ × length × width^2^. On day 24, the tumours were excised and photographed. The data were shown as mean with SD of four mice in each group. B, Immunohistochemistry staining of nude mice tumour tissue sections for EGFR, p‐EGFR, N‐cadherin and vimentin were shown. C, Immunohistochemistry staining of citH3, p‐EGFR E‐cadherin in PDAC patients. D, Relationship between CitH3, p‐EGFR and E‐cadherin expression in 150 PDAC patients’ tissues

To examine the effects of NETs on migration and invasion of pancreatic cancer in vivo, we injected MIA PaCa2 cells subcutaneously into the ventral flanks of 6‐week‐old female BALB/c nude mice. MIA PaCa2 cells were pretreated with different types of CM. Consistent with in vitro experiments, NETs did not affect tumour growth in vivo (Figure 6A). But the immunostaining results showed that MIA PaCa2 treated with CM‐NETs exhibited high expression of N‐cadherin and vimentin companied with activation of EGFR phosphorylation (Figure 6B).

To further investigate whether NETs lead to EMT by promoting EGFR phosphorylation in patient specimens, we evaluated NETs, pEGFR and E‐cadherin expression levels in 150 PDAC samples. The immunohistochemical staining results of PDAC tumour samples showed that the existence of NETs was positively correlated with pEGFR expression and negatively correlated with E‐cadherin expression (Figure 6C, D). The data collectively showed that the presence of NETs correlated with EGFR activation and EMT in patients with PDAC.

## 4 DISCUSSION

Recent researches have highlighted the importance of signalling crosstalks between the microenvironment and cancer, especially between cancer cells and immune cells.^44^ Previous studies suggest that NETs play important roles in cancer metastases via multiple mechanisms.^45^ Intravascular NETs can promote extravasation of cancer cells by increasing local vascular permeability.^10^ Another study has suggested that NETs could promote both the invasion and expansion of cancer cells by concentrating proteases and protecting them from endogenous inhibitors.^11^ We have found a new pathway that NETs promote tumour migration and invasion in pancreatic cancer.

Growing evidences suggest that EMT plays important roles in the invasion and metastasis of PDAC. In our study, pancreatic cancer cells showed an enhanced ability of migration and invasion following treatment with NETs. Further, results showed that NETs from both PDAC patients and normal individuals could induce EMT. EMT is believed to be important in the development of pancreatic cancer.^30^ Here, we showed that NETs induced morphological changes related to EMT in pancreatic cancer cells for the first time. We will perform more experiments to investigate how NETs promote EMT in pancreatic cancer cells.

To determine the mechanism by which NETs promote EMT, we utilized Western blot analyses to detect several important pathways related to EMT. The results showed that the EGFR/ERK pathway was activated and essential in NETs‐induced EMT. EGFR inhibitors such as gefitinib, lapatinib and erlotinib, have been widely used clinically in several cancers such as NSCLC.^46^ Our study provides a new thought on targeted therapy for PDAC patients with NETs.

IL‐1β is of great importance in inflammation and could stimulate cancer malignant potential. It has been reported that IL‐1β could induce EMT in multiple cell types and disease states. IL‐1β promoted EMT by activating TGF‐β1 in human bronchial epithelial cells,^47^ by inducing methylation of the oestrogen receptor ERα gene in breast cancer cells^48^ and by mediating EMT via the FGF‐2 pathway.^49^

Our study showed inhibition of IL‐1β signalling by antibodies markedly blocked EGFR/ERK‐dependent EMT induced by NETs. It has been reported that NETs can serve as platforms for the processing and activation of IL‐1 family cytokines, and NETs can potently process and activate IL‐1α and IL‐36 subfamily cytokines.^50^ Our study is the first time to show that NETs derived from both PDAC patients’ and normal individuals’ neutrophils could both produce IL‐1β.

In our study, anti‐IL‐1β antibodies abolished NETs‐induced EMT by blocking the EGFR/ERK pathway. It has been reported that EGFR affects the progression of pancreatic cancer through a variety of signalling pathways.^51^ And, the inhibition of the formation of NETs and the blockade of IL‐1β can effectively attenuate the EGFR‐induced progression of pancreatic cancer. A previous study showed that IL‐1β could activate EGFR via the CXCL1‐CXCR2 axis in oral cancers.^51^ It has also been reported that IL‐1 drives breast cancer growth and bone metastasis in vivo.^52^ Pharmacological blockade of IL‐1 receptor (IL‐1R) decreased the primary growth of 4T1 tumours and reduced the systemic levels of myeloperoxidase, cell‐free DNA (cfDNA) and G‐CSF, without interfering with neutrophil counts. Thus, the inhibition of NETs/ IL‐1β/EGFR signalling is a new hope for targeted therapy of pancreatic cancer.^53^ Importantly, blocking IL‐1 with IL‐1 antagonist (IL‐Ra) inhibited tumour growth and metastasis accompanied by decreased myeloid cell accumulation.^54^ IL‐1β is reported to play important roles in vivo. In the further study, we will perform more experiments in vivo to investigate the interaction between NETs and pancreatic cancer.

Neutrophils can exhibit a pro‐tumourigenic N2 phenotype and an anti‐tumourigenic N1 phenotype. No specific marker has been identified to distinguish the N1/N2 phenotypes clinically until now.^55^ Due to the difficulty in distinguishing neutrophil subtypes, it is very difficult to develop a drug to target N2 neutrophils. NETs are generated from activated neutrophils, but no phenotypic subtypes have been described in NETs to date.^56^ Thus, NETs may be easier than neutrophils to identify in pathological tissue and serve as a possible target for PDAC therapy.

In general, NETs promote pancreatic cancer EMT, migration and invasion dependent on EGFR/ERK pathway through IL‐1β, providing new thoughts on the interaction between NETs and pancreatic cancer cells. Thus, the study of NETs may provide new biomarkers for PDAC.

## CONFLICT OF INTEREST

The authors confirmed that there was no conflict of interest.

## AUTHORS’ CONTRIBUTION

**Wei Jin:** Formal analysis (equal); Resources (equal); Validation (equal); Writing‐original draft (equal); Writing‐review & editing (equal). **Liang Liu:** Investigation (equal); Resources (equal); Supervision (equal). **Huaxiang Xu:** Data curation (equal); Funding acquisition (equal); Resources (equal); Visualization (equal). **Huijing Yin:** Conceptualization (equal); Investigation (equal); Methodology (equal). **Hao Li:** Conceptualization (equal); Investigation (equal). **Xianjun Yu:** Supervision (equal).

## Supporting information

Fig S1‐S4

## Data Availability

The data that support the findings of this study are available from the corresponding author upon reasonable request.
