# Supplementary material for: Neutrophil extracellular DNA traps promote pancreatic cancer cells migration and invasion by activating EGFR/ERK pathway
Source: J Cell Mol Med. 2021 May 6;25(12):5443–56. doi: 10.1111/jcmm.16555 (PMC8184670; doi:10.1111/jcmm.16555)
Supplement: Supplementary file 1 — Fig S1‐S4 [file JCMM-25-5443-s001.docx]

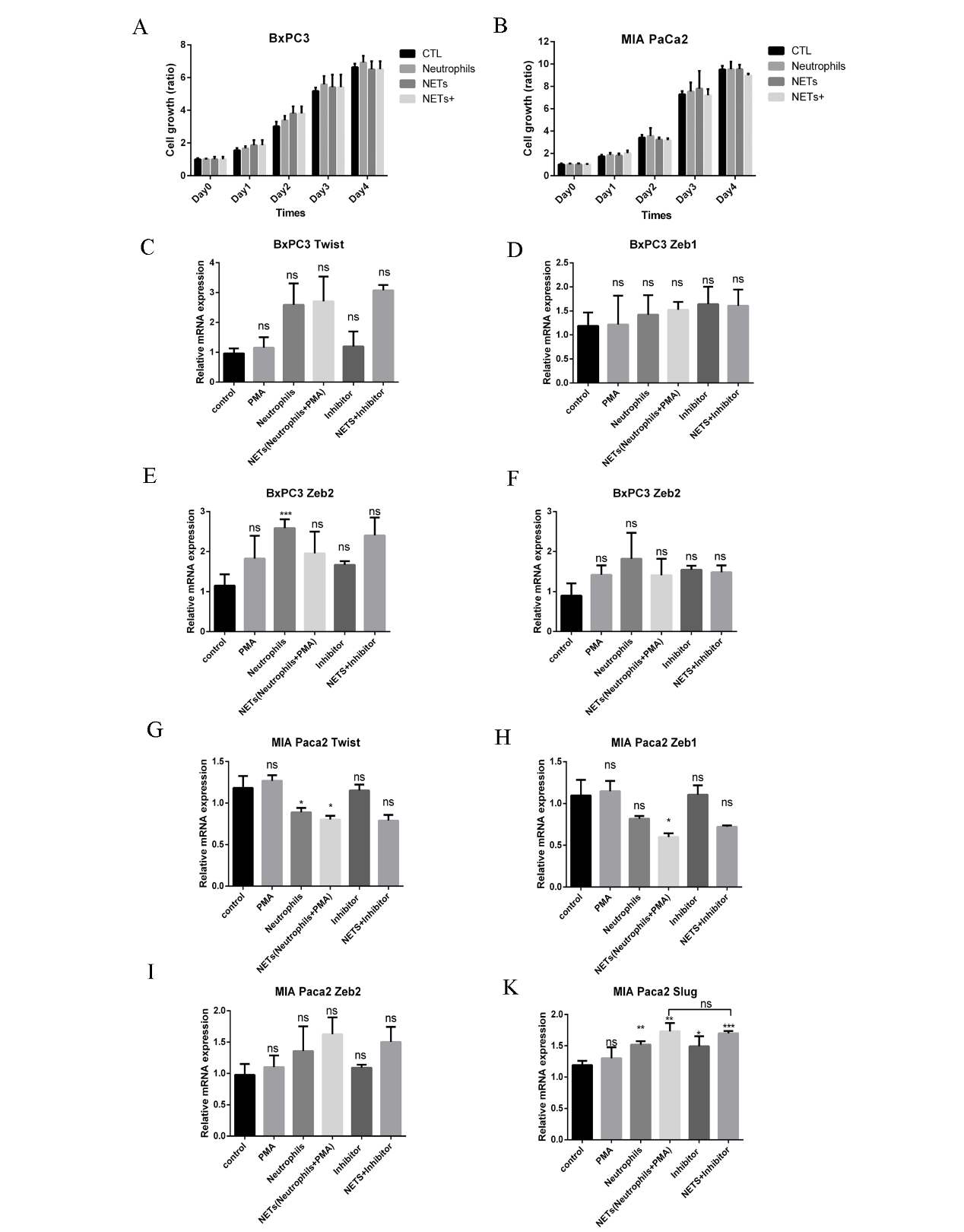


Figure S1

A. CCK8 assays were applied to analyze the proliferation of BxPC3 for indicated days. B. CCK8 assays were applied to analyze the proliferation of MIA PaCa2 for indicated days. C-F. RT-qPCR was carried out to identify the mRNA level of Twist, Zeb1, Zeb2, Slug in BxPC3 cells. G-J. RT-qPCR was carried out to identify the mRNA level of Twist, Zeb1, Zeb2, Slug in MIA PaCa 2cells.


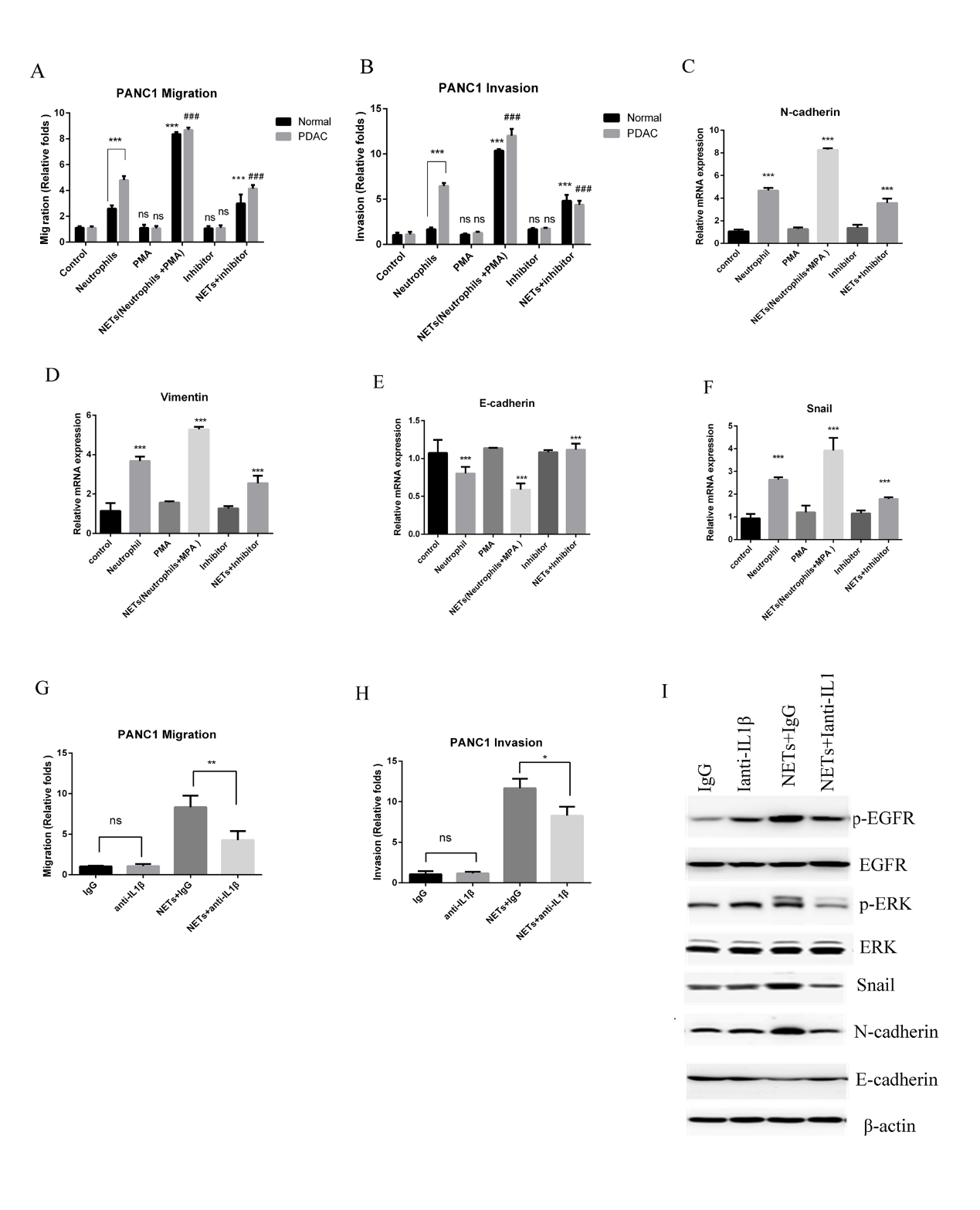


Figure S2

A,B Neutrophils were extracted from the blood of PDAC patients or normal human blood , and the formation of NETs were induced. The migration and invasion assay were carried out in PANC1 cells with indicated conditioned medium. The neutrophils and NETs conditioned medium were added to the lower chamber. 1x10^5^ PANC1 were added to the upper chamber without serum. The concentrations of PMA and sivelestat are 5nM and 10nM . The number of cells passing through the upper chamber were counted in three fields 48 hours later (magnification, × 20). C-F PANC1 cells were co-cultured for 24 h with conditioned medium. Realtime-PCR was used to identify the expression of indicated genes in PANC1. G-H. The migration and invasion ability of PANC1 cells were tested by Wound-healing and Invasive Transwell assay with the treatment of IL1β antibody. I.PANC1 cells were treated with indicated condition for 48 hours .Western blot was carried out to identify the indicated proteins levels, β -actin was used as the loading control.


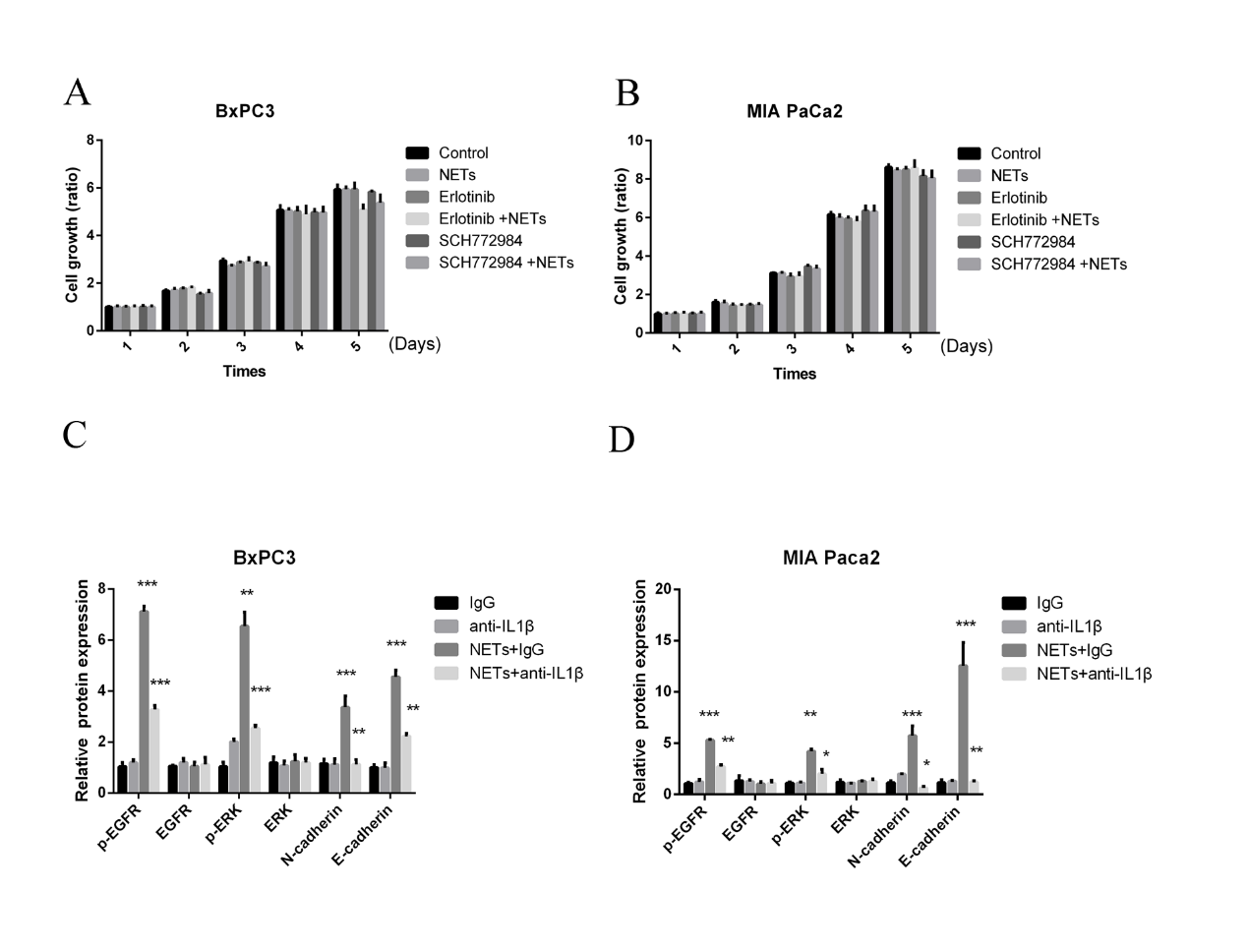


Figure S3

A.CCK8 assays were applied to analyze the proliferation of BxPC3 for indicated days. B. CCK8 assays were applied to analyze the proliferation of MIA PaCa2 for indicated days. C-D. Relative expression of the protein was calculated by Image J. β -actin was used as the loading control. Results are presented as the mean ± SD(n=3).


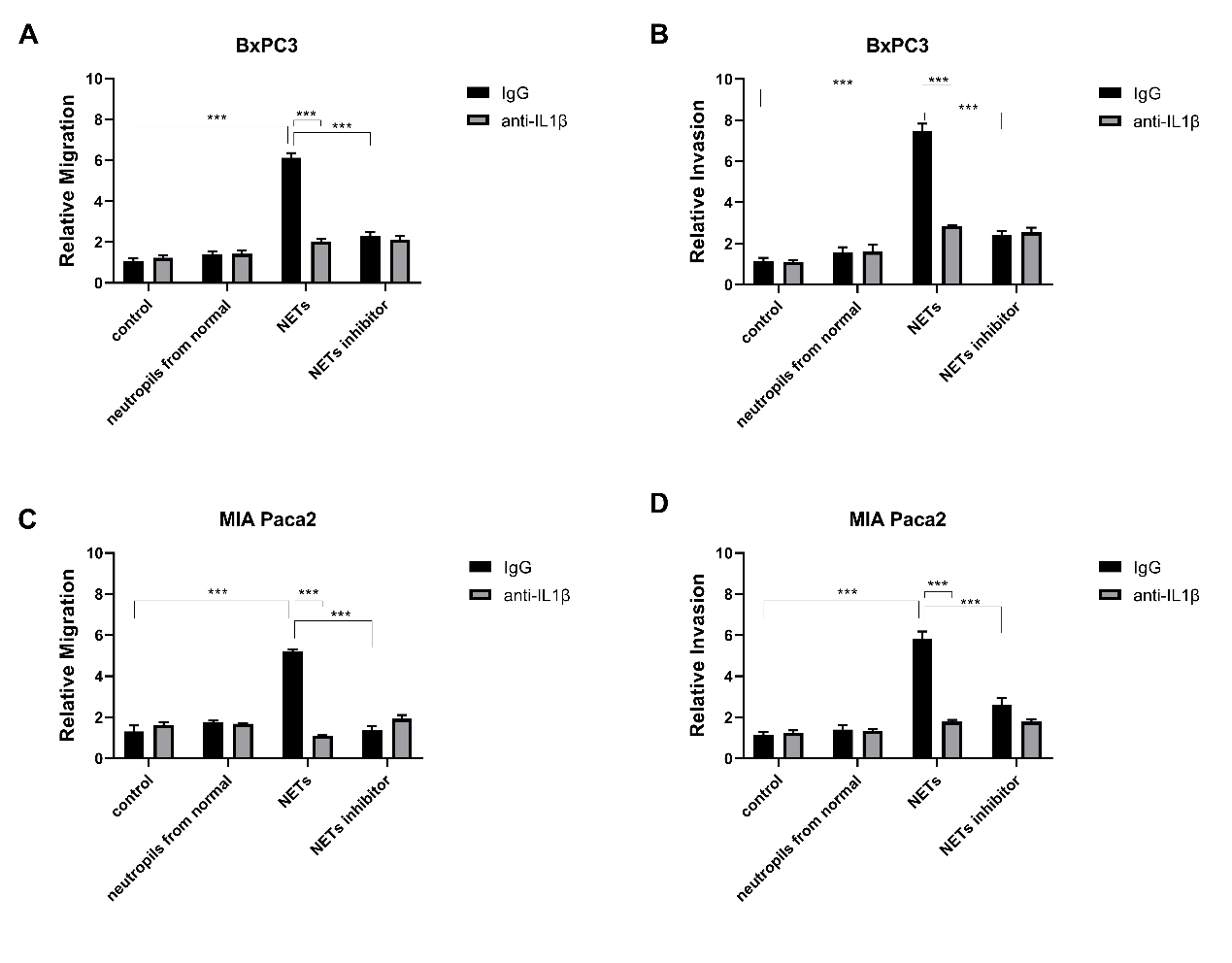


Figure S4.

Blockage of IL1β could effectively inhibit NETs induced migration and invasion in pancreatic cancer cells.A. Migration assay in BxPC3 cell line. B. Invasion assay in BxPC3 cell line. C. Migration assay in MIA Paca2cell line. D. Invasion assay in MIA Paca2 cell line.
